# Global Motion Perception in Autism Spectrum Disorder: A Meta-Analysis

**DOI:** 10.1007/s10803-019-04194-8

**Published:** 2019-09-06

**Authors:** Ruth Van der Hallen, Catherine Manning, Kris Evers, Johan Wagemans

**Affiliations:** 1 Laboratory of Experimental Psychology, Department of Brain and Cognition, KU Leuven, 3000 Leuven, Belgium; 2 Leuven Autism Research (LAuRes), KU Leuven, 3000 Leuven, Belgium; 3 Clinical Psychology, Department of Psychology, Education & Child Studies, Erasmus University Rotterdam, 3062 PA Rotterdam, The Netherlands; 4 Department of Experimental Psychology, University of Oxford, Oxford, OX2 6GG UK; 5 Parenting and Special Education Research Unit, KU Leuven, Leuven, 3000 Belgium

**Keywords:** Autism spectrum disorder (ASD), Meta-analysis, Motion perception, Biological motion, Coherent motion

## Abstract

Visual perception in individuals with autism spectrum disorder (ASD) is often debated in terms of enhanced local and impaired global perception. Deficits in global motion perception seem to support this characterization, although the evidence is inconsistent. We conducted a large meta-analysis on global motion, combining 48 articles on biological and coherent motion. Results provide evidence for a small global motion processing deficit in individuals with ASD compared to controls in both biological and coherent motion. This deficit appears to be present independent of the paradigm, task, dependent variable, age or IQ of the groups. Results indicate that individuals with ASD are less sensitive to these types of global motion, although the difference in neural mechanisms underlying this behavioral difference remains unclear.

## Introduction

Autism spectrum disorder (ASD) is an early-onset neurodevelopmental condition affecting approximately 1% of the population. ASD is characterized by a co-occurrence of impairments in social reciprocity and social communication, and rigid, repetitive patterns of behavior, interest or activities (DSM-5; American Psychiatric Association 2013). Whereas the condition is best-known for its profound impact on the social domain, its impact on the non-social domain, which includes atypical responses to sensory input or unusual interests in sensory aspects of the environment, has gained increased recognition over the last decade (APA 2013; Robertson and Simmons 2015). As such, sensory atypicalities, like hypersensitivity to light (i.e., lights seem overly bright) or hyposensitivity to sounds (i.e., seemingly ignoring someone calling your name) have been found to carry wide-ranging effects on everyday life, including family life and education (e.g., Ashburner et al. 2008; Bagby et al. 2012; Robertson and Simmons 2015).

One sensory modality where atypicalities in ASD have been particularly well-studied is vision (see Simmons et al. 2009 for a review). Here, we focus on an important aspect of atypical visual processing in ASD which has received much attention: global motion processing. Local motion processing involves sensitivity to the direction of motion in a small region of an image and depends on neurons with small directional receptive fields in area V1 (see Movshon 1990 for a review). Global motion processing, obtained through integration of local motion signals across space (Smith et al. 1994), depends on areas further along the dorsal stream, primarily the MT/MST complex and a number of extrastriate areas and areas in the intraparietal sulcus (Baker et al. 1991; see Hadad et al. 2015 for an overview). Motion perception lies at the interface between perception and action, allowing individuals to track and/or grasp objects and navigate within a dynamic world. As a result, atypical global motion processing (Kaiser and Shiffrar 2009) will have marked effects on how an individual perceives and interacts with the world. Some studies have suggested altered or disturbed global motion processing in ASD, although the overall evidence is mixed (Kaiser and Shiffrar 2009).

There are a number of neurocognitive theories that aim to explain why global motion processing may be atypical in individuals with ASD. Some scholars have proposed theories that postulate specific difficulties with processing motion or moving information. For example, it has been suggested that motion processing may be disproportionally impaired in a range of developmental conditions, including ASD, due to an increased vulnerability of the dorsal (Braddick et al. 2003) or magnocellular (Greenaway et al. 2013) pathways. Along the same lines, it has been suggested that motion processing in ASD may be affected due to poor integration of information over space and time (Gepner and Féron 2009). Other scholars have proposed domain-general theories of ASD where more general impairments may, amongst other abilities, affect motion processing abilities as well. For example, it has been suggested that individuals with ASD focus more on details within a visual scene, either due to enhanced processing of local details (Mottron et al. 2006) and/or a failure to integrate information (Happé and Frith 2006) in order to perceive the overall whole. As a result, one could expect individuals with ASD to show particularly pronounced atypicalities in tasks that require complex integration of motion information in order to form a global percept.

Much of the global motion research has focused on two motion paradigms in particular, namely *coherent motion* and *biological motion*, as both these paradigms rely heavily on integrating local motion information into a global motion pattern (Kaiser and Shiffrar 2009). In a standard coherent motion paradigm, which uses random-dot kinematograms (RDKs), observers are required to integrate individual local motions into a global coherent motion. The stimuli are made up of two populations of moving dots: a percentage of dots moving “coherently” over time, (i.e., signal dots) and a percentage of dots moving in random directions (i.e., noise dots) (Newsome and Paré 1988). Important stimulus parameters include the density, speed and lifetime (how long each dot persists on the screen) of the dots used. The task at hand cannot be performed by processing single dots alone, but requires integration across the local motion signals, and is thus assumed to reflect global motion processing. Accordingly, motion coherence paradigms activate areas of the brain involved in integrating motion signals, such as area MT/V5 (Britten et al. 1992; Tootell et al. 1995). Sensitivity to coherent motion is typically assessed by measuring the observers’ coherence threshold: the minimum signal-to-noise ratio required to detect coherent motion or discriminate motion direction at a predefined performance level.

The first study to assess motion coherence thresholds in ASD was conducted by Spencer et al. (2000). Here, the authors reported that children with ASD required a higher proportion of coherently moving dots in order to perceive the overall motion, compared to typically developing (TD) children. While this finding of elevated motion coherence thresholds in ASD has been replicated many times (e.g., Koldewyn et al. 2010; Milne et al. 2002; Pellicano et al. 2005), other studies have failed to replicate the result, instead finding comparable thresholds between individuals with ASD and TD individuals (e.g., Brieber et al. 2010; Del Viva et al. 2006; Jones et al. 2011; Koldewyn et al. 2013; Milne et al. 2006; Price et al. 2012).

In a standard biological motion paradigm, which uses point light animation displays (PLDs), observers are required to perceive the motion of a living form (most typically, a human figure carrying out a particular action such as walking or dancing) constructed of moving points of light (Johansson 1973). The paradigm requires integration across these points of light in order to disambiguate the form. The PLDs can differ with regard to the number, speed and position of dots, as well as the duration and type of the displayed motion. As a control condition, paradigms on biological motion often include a condition where the original motion trajectories are scrambled to create non-biological motion with the same local motion signals. The perception of biological motion has been shown to activate a network of areas in the extrastriate cortex, in particular the posterior superior-temporal sulcus (pSTS; Grossman and Blake 2002; Grossman et al. 2000) and areas that receive input from both the dorsal and ventral streams, as well as the ventral premotor cortex (vPMC; Saygin 2007; for an overview, see Hadad et al. 2015). Sensitivity to biological motion is typically assessed by measuring the percentage correct (accuracy) or reaction time necessary for observers to detect biological motion or discriminate between certain types of motion.

The first study to investigate sensitivity to biological motion in ASD was conducted by Moore et al. (1997). Moore et al. reported that individuals with ASD were able to distinguish human forms from inanimate objects and recognize actions portrayed by human forms similarly to those without ASD, but showed difficulties in discriminating attitudes and states portrayed in the displays. Later, Blake et al. (2003) reported that individuals with ASD showed basic impairments in distinguishing biological motion from phase-scrambled motion displays compared to typical individuals. Mirroring the motion coherence literature, replication attempts on biological motion have yielded mixed results, with some studies finding reduced sensitivity to biological motion in ASD (Annaz et al. 2009; Blake et al. 2003; Koldewyn et al. 2010; Koldewyn et al. 2011; Nackaerts et al. 2012; Price et al. 2012; Rutherford and Troje 2012) or a lack of preferential looking for biological motion versus phase-scrambled motion (Annaz et al. 2012; Chaminade et al. 2015; Crawford et al. 2016; Falck-Ytter et al. 2013; Franchini et al. 2016; Klin et al. 2009; Wang et al. 2015; Wright et al. 2016), and others finding no differences between individuals with and without ASD (Cusack et al. 2015; Hubert et al. 2006; Jones et al. 2011; Murphy et al. 2009; Saygin et al. 2010). However, results have been consistent in showing a reduced ability in individuals with ASD compared with TD individuals to extract emotional content from biological motion displays (e.g., Hubert et al. 2006; Moore et al. 1997; Nackaerts et al. 2012; Parron et al. 2008).

What might account for the discrepancies in these results for motion coherence and biological motion in ASD? A number of studies have revealed considerable individual variability in performance, with only a subset of individuals with ASD showing elevated thresholds in motion paradigms (Milne et al. 2002, 2006; Pellicano and Gibson 2008; Takarae et al. 2008). Mixed findings at the group level could therefore be the result of a sampling error. In reviewing the existing evidence, however, it becomes clear that differences in participant, stimulus, task and/or paradigm characteristics are likely to play an active role in explaining the discrepancies (see Kaiser and Shiffrar 2009; and Simmons et al. 2009 for a discussion). For example, differences in global motion processing have been linked to participant characteristics such as chronological age, verbal and non-verbal ability, and language delays (Jones et al. 2011; Koldewyn et al. 2010; McKay et al. 2012; Rutherford and Troje 2012; Takarae et al. 2008) as well as stimulus characteristics such as stimulus duration (Robertson et al. 2012; but also see Davis et al. 2006), presentation location (Ronconi et al. 2012) and stimulus speed (Manning et al. 2013). Yet, it is difficult to quantify the potential impact of these characteristics by merely comparing or reviewing individual studies, given the range of different participant groups, stimuli, tasks and paradigms that have been used. It is, however, of critical importance to understand these atypicalities in coherent motion and biological motion in order to evaluate theories of perception in ASD.

Therefore, this study takes a meta-analytic approach to assess the existing evidence on coherent motion and biological motion in individuals with ASD relative to TD individuals. This approach, interesting due the large number of studies using these two paradigms in participants with ASD, allows us to systematically integrate findings across studies, to conduct hypothesis-testing regarding sources of heterogeneity, and to quantify biases. The moderators included in this meta-analysis pertain to task (e.g., type of paradigm, discrimination vs. detection or sensitivity vs. ability), stimulus (e.g., stimulus duration or stimulus speed) and participant characteristics (e.g., age, gender or IQ), and include those variables that are most frequently reported in the global motion literature or have been suggested to rule the variability and contradictions in the present data (see below).

## Methods

### Sample of Studies

In order to find eligible studies, a literature search was conducted using two supplementary search strategies. Firstly, a computerized search was conducted using a well-designed Boolean operation that combined ASD, motion processing and vision as the three key components: (“autis*” OR “ASD” OR “ASC” OR “asperger*” OR “PDD*”) AND (“motion” OR “mov*”) AND (“vis*” OR “perc*”). We covered a wide time span (January 1980–August 2018), exploring titles, abstracts and keywords, searching both Web of Science and PubMed electronic databases. In total, 1460 unique hits were obtained. Secondly, a manual literature search was performed on the reference and citation lists of ten review or primary study articles in order to find studies that might be relevant but were not yielded in the computerized search. This manual search brought forth one additional study.

**Fig. 1:**
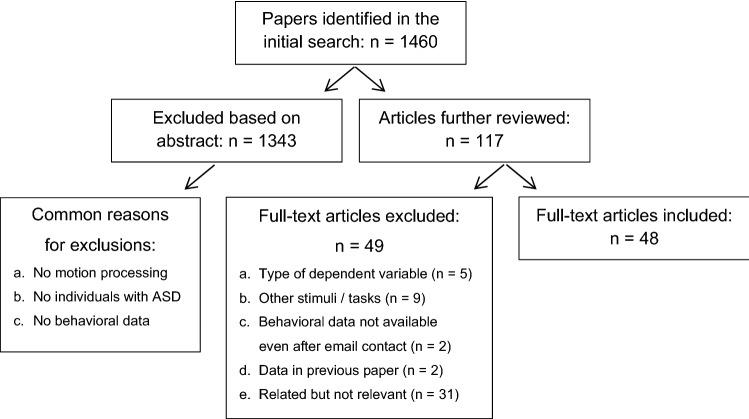
Selection process. This flowchart displays the entire in- and exclusion process of gathering articles to be included in the meta-analysis

The selection and exclusion process of all abstracts yielded in the search was done by three researchers, including the first author. An overview of the selection and exclusion process is shown in Fig. 1. Interrater agreement on in- or exclusion of articles was checked for the first 200 abstracts, and resulted in a Fleiss’ Kappa of 89%. The remaining abstracts were divided amongst the three raters and evaluated by at least one of the raters. In case of doubt or ambiguity, the abstract was discussed amongst the three researchers. Exclusion of research material was mostly related to fact that the paper (1) did not discuss motion processing, (2) did not administer the task to individuals with ASD (but used ASD-relatives or typically developed individuals with ASD traits), and/or (3) did not report any behavioral data. Data that focused on the ability to extract social or emotional content from motion displays (e.g., Hubert et al. 2006; Moore et al. 1997; Nackaerts et al. 2012; Parron et al. 2008) were not included. The rationale behind this decision was that the social or emotional content adds an extra level of difficulty to global motion tasks and no longer allows us to extract a pure measure of one’s global motion perception ability. Observers may perceive global motion accurately, but fail to perform an adequate social and/or cognitive analysis of the perceptual information, impacting their overall performance.

The in- and exclusion process resulted in a set of 48 individual articles that evaluated coherent motion (*N* = 28, see Table 1 for an overview) or biological motion (*N* = 20, see Table 2 for an overview) in individuals with ASD and a TD control group. Authors were contacted in case relevant information was missing or included in a format that was not directly accessible (e.g., summary data merely presented in graphs or figures).

**Table 1:** A summary of studies on coherent motion in ASD included in the meta-analysis

	Study	ASD		TD		Task type	Stimulus duration	Number of dots	Speed (deg/s)	Diameter (deg)	Lifetime (ms)	Dot density (dots/deg^2^)	Results
		Age	N	Age	N								
1	Annaz et al. (2009)	5–12 years	23	4–12 years	34	CM detection	NS	NS	3.21	NS	NS	NS	ASD < TD
2	Atkinson (2009)	18–58 years	13	17–54 years	16	CM discrimination	200	750	2.0^a^	~1 mm	NS	5.9^a^	ASD < TD
3	Brieber et al. (2010)					CM discrimination	1000	300	2.5				
4	Chen et al. (2012)	13–18 years	19	13–18 years	17	CM discrimination	300	200	5.25	0.03	Unlimited	5.19^a^	ASD = TD
5	David et al. (2014)	24–45 years	15	24–45 years	14	CM discrimination	750	N/A	2.4–3.0	0.36	N/A	N/A	ASD = TD
6	Davis et al. (2006)	10–18 years	9	7–15 years	9	CM discrimination	220, 1000	100	6.36	0.1	NS	2.52^a^	ASD = TD (200 ms) ASD < TD (1000 ms)
7	de Jonge et al. (2007)	7–33 years	29	7–33 years	32	CM detection	NS	NS	NS	1 pixel	NS	NS	ASD = TD
8	Del Viva et al. (2006)	6–14 years	10	6–7 years 8–12 years	12 14	CM discrimination	160	100	10	0.1	66	0.44^a^	ASD = TD
9	Foss–Feig et al. (2013)	8–17 years	15	8–17 years	17	CM discrimination	NS	N/A	4	1, 2.5, 6	NS	NS	ASD = TD (HC) ASD > TD (LC)
10	Greimel et al. (2013)	9–16 years	17	8–15 years	17	CM discrimination	420	300	5	0.038	NS	3.12	ASD = TD
11	Jones et al. (2011)	14–16 years	89	14–16 years	52	CM detection	<6000	NS	2.5	0.23	40	NS	ASD = TD
12	Koldewyn et al. (2013)	5–12 years	28	5–12 years	26	CM discrimination	1000	100	11	0.2	50	0.64^a^	ASD = TD
13	Koldewyn et al. (2010)	11–19 years	30	11–19 years	32	CM discrimination	2000	NS	4.5–9	NS	NS	NS	ASD < TD
14	Manning et al. (2013)	7–13 years	28	7–14 years	32	CM detection	1000	200	1.5, 6	0.34	83	0.83	ASD < TD (slow) ASD = TD (fast)
15	Manning et al. (2015)	7–13 years	31	7–13 years	31	CM detection	1000	200	1.5	0.34	83, Unlimited	0.83	ASD = TD
16	Manning et al. (2015)	6–13 years	33	6–13 years	33	CM discrimination	400	100	1.5, 6	0.44	Unlimited	NS	ASD > TD (noise) ASD = TD (no noise)
17	Milne et al. (2002)	9–15 years	25	9–15 years	22	CM discrimination	1010	150	8.8	1 pixel	224	0.3	ASD < TD
18	Milne et al. (2006)	8–12 years	23	8–12 years	23	CM detection	2300	600	7	0.1	85	2.14	ASD = TD except for a particular subgroup
19	Peiker et al. (2015)	24–45 years	13	23–46 years	14	CM discrimination	750	NS	2.4–3.0	0.36	NS	NS	ASD = TD
20	Pellicano and Gibson (2008)	8–12 years	20	8–12 years	20	CM discrimination	600	100	6.3	0.1	30	0.4	ASD < TD
21	Price et al. (2012)	7–23 years	14	7–23 years	16	CM detection	2300	1200	7^a^	0.1	200	2.1^a^	AD = TD
22	Robertson et al. (2012)	24 (3.5) years	20	30 (11.7) years	20	CM discrimination	200, 400, 1500	150	5	0.04	~ 50^a^	1.85	ASD < TD (200 ms) ASD = TD (400, 1500 ms)
23	Robertson et al. (2014)	16–27 years	18	15–23 years	18	CM discrimination	200, 600	150	5	0.04	~ 50^a^	1.85	ASD < TD (200 ms) ASD = TD (600 ms)
24	Ronconi et al. (2012)	9–18 years	11	11–18 years	11	CM discrimination	300	NS	12	0.05, 0.15	~ 50	17	ASD < TD (central) ASD = TD (peripheral)
25	Spencer and O’Brien (2006)	14 (3.3) years 12 (3.6) years	15 10	12 (2.4) years	15	CM detection	250	N/A	5.8	NS	50	4	ASD < TD AS = TD
26	Spencer et al. (2000)	7–11 years	23	7–11 years	50	CM detection	Self–timed	2000	5.8	NS	17^a^	4	ASD < TD
27	Tsermentseli et al. (2007)	17–40 years	21	17–40 years 17–40 years	20 20	CM detection	250	N/A	5.8	NS	50	4	ASD < TD AS = TD
28	Yamasaki et al. (2011)	20–39 years	12	20–39 years	12	CM discrimination	750	400	5	0.20	NS	0.16	ASD = TD

*CM* coherent motion, *ASD* individuals with ASD,* AS* individuals with Asperger Syndrome, *TD* typically developing individuals, *NS* not specified, *HC* high contract, *LC* low contrast, *N/A* not applicable

^a^Information not explicitly stated, but calculated from the information that was provided

**Table 2:** A summary of studies on biological motion in ASD included in the meta-analysis

	Study	ASD		TD		Task type	Type of motion	Stimulus duration (ms)	Number of dots	Speed (deg/s)	Results
		Age	N	Age	N						
1	Blake et al. (2003)	8–10 years	16	5–10 years	9	BM detection	Human body actions	1000	9	4	ASD < TD
2	Van Boxtel et al. (2016)	8–18 years	16	8–18 years	17	BM discrimination	Human body actions	1000	13	2 steps/s	ASD < TD
3	Cleary et al. (2014)	11–17 years	13	12–15 years	13	BM detection	Human gait	NS	13	NS	ASD = TD
4	Cook et al. (2009)	34 (12.4) years	16	33 (12.2) years	16	BM detection	Human versus object motion	NS	1	NS	ASD < TD
5	Cusack et al. (2015)	16 (2.2) years	18	16 (2.2) years	18	BM detection BM discrimination	Human body gait, actions	1500, 2500	12	NS	ASD = TD
6	Freitag et al. (2008)	18 (3.6) years	13	19 (1.2) years	15	BM detection	Human gait	1500	15	NS	ASD = TD
7	Herrington et al. (2007)	28 (7.1) years	10	26 (4.9) years	10	BM detection	Human gait	1000	13	NS	ASD = TD
8	Hubert et al. (2006)	15–34 years	19	15–34 years	19	BM discrimination	Human body actions, objects	5000	5–10	NS	ASD = TD
9	Jones et al. (2011)	14–17 years	89	14–17 years	52	BM detection	Human gait	6000	11	NS	ASD = TD, except for low IQ
10	Koldewyn et al. (2010)	11–20 years	25	12–20 years	25	BM detection	Human gait	2000	13	4.5–9	ASD < TD
11	Kröger et al. (2014)	6–15 years	17	6–15 years	21	BM detection	Human gait	1000	15	2 steps/s	ASD = TD
12	McKay et al. (2012)	18–38 years	10	19–37 years	10	BM detection	Human gait	1000	15	NS	ASD = TD
13	Murphy et al. (2009)	26 (7.8) years	16	26 (2.9) years	16	BM detection	Human gait	6500	11	NS	ASD = TD
14	Nackaerts et al. (2012)	35 (8.5) years	12	32 (6.3) years	12	BM detection	Human gait and actions	3000	12	NS	ASD < TD
15	Parron et al. (2008)	7–18 years	23	12 (2.4) years	23	BM discrimination	Human body and objects	5000	5–10	NS	ASD = TD
16	Price et al. (2012)	7–23 years	14	7–24 years	16	BM discrimination	Human gait, legs and feet	5000	6	NS	ASD < TD
17	Rutherford et al. (2012)	29 (6.0) years	13	31 (9.0) years	14	BM detection BM discrimination	Human gait	1000	11	NS	ASD = TD
18	Saygin et al. (2010)	34 (12) years	16	34 (11) years	20	BM detection	Human gait and objects	580	12	NS	ASD = TD
19	Swettenham et al. (2013)	10 (1.5) years	14	9 (1.8) years	14	BM discrimination	Human gait	2000	13	NS	ASD = TD
20	Weisberg et al. (2014)	12–21 years	19	13–19 years	19	BM detection	Human gait and object	2500	9	NS	ASD = TD

*BM* biological motion, *ASD* individuals with ASD, *TD* typically developing individuals, *NS* not specified

### Coding

All 48 articles were coded by one of three researchers, including the first author. All coded data were later double-checked by the first author. Data extraction included study descriptors (e.g., author names and year of publication), participant demographics (e.g., sample size, chronological age, gender and IQ), ASD diagnostic procedures, composition of the comparison group, and type of group-matching, as well as details regarding the experimental task, such as the type of paradigm (i.e., coherent motion vs. biological motion), type of task (detection vs. discrimination), stimulus details (e.g., motion speed, number of dots and dot size) and descriptive statistics (means and standard deviations) of the study findings. When the descriptive statistics were not available, information on the test statistics (e.g., *t*- or *r*-values) was collected.

### Moderator Variables

The moderators included in this meta-analysis pertain to task, stimuli and participant characteristics. In deciding which moderator variables to focus on, we considered both the extent to which the variables have been reported in the existing literature as well as the degree to which variables have been suggested with regard to variability within the existing data.

With regard to task characteristics, the type of paradigm (biological motion vs. coherent motion) the type of task (motion discrimination vs. motion detection) and type of dependent variable (accuracy, RT data, threshold or d-primes) were retained for further analysis. The type of paradigm, i.e. biological motion vs. coherent motion, may be the most obvious characteristic to include. Both biological motion and coherent motion rely on the integration of local motion into a global motion pattern and both are considered prime global motion processing paradigms. However, partially different neural network areas are thought to be involved (Saygin 2007; Grossman and Blake 2002; Grossman et al. 2000; Britten et al. 1992; Tootell et al. 1995), suggesting either paradigms may constitute unique challenges. In addition, it has been argued that the *social* layer of biological motion makes it a more high-level paradigm, and perhaps more difficult than the coherent motion paradigm, especially for individuals with ASD (Kaiser and Shiffrar 2009). The type of task, i.e., motion detection versus motion discrimination, has received far less attention in the ASD literature. In a motion detection task, participants are asked to detect global motion patterns (e.g., detect biological vs. non-biological motion or detect coherent vs. non-coherent motion), while in a motion discrimination task, participants are asked to discriminate between different types of global motion patterns (e.g., different types of PLD or different directions of coherent motion). In other words, while for motion detection the question is whether participants can distinguish between global motion patterns and random motion, for motion discrimination the question is to what (fine-grain) extent participant can distinguish between different levels or different types of global motion pattern. As such, one could argue that detecting the presence of global motion (compared to *noise* or random motion) is a more *basic* task compared to discriminating between different types of global motion. Previous research has indicated that different mechanisms and different neuronal populations are involved in motion detection compared to motion discrimination (Hol and Treue 2001; Koyama et al. 2010), highlighting its potential as moderator of the effect. Research in typically developing individuals that compared thresholds for a detection versus discrimination paradigm using flickering gratings (McCarthy et al. 1994) or investigated spatial offsets with Vernier stimuli (Harris and Fahle 1995) suggests this distinction can be important. As final task characteristic, the type of dependent variable was considered. Most global motion studies record either accuracy, RTs, motion thresholds, accuracy, or d-primes. However, while all four are valid outcome measures, they do tap into different aspects of one’s global motion perception performance, i.e., some people can run, some people are fast runners, and some people are good long-distance runners. As differences in visual performance tasks between individuals with and without ASD are known to depend on the dependent variable in question, i.e., comparable accuracy but longer RT’s for global processing in individuals with ASD compared with TD individuals (e.g., Van der Hallen et al. 2015), the type of dependent variable was considered as moderator of the effect.

As for stimulus characteristics a wide range of parameters (i.e., speed, size, surface area, stimulus duration, etc.) was coded, both for biological motion and coherent motion. Previous studies have actively investigated a number of stimulus parameters in the hopes of explaining the discrepancies in the existing findings. For example, elevated motion coherence thresholds have been found in individuals with ASD specifically for stimuli that were (1) presented at short but not long durations (Robertson et al. 2012; but also see Davis et al. 2006), (2) presented centrally but not peripherally (Ronconi et al. 2012) and (3) presented at slow but not fast stimulus speeds (Manning et al. 2013). These studies have particular value as they each investigated the effect of these stimulus parameters within the same set of individuals, whilst controlling other variables, providing evidence for the importance of these parameters. Interestingly, a recent meta-analysis on coherent motion processing in dyslexia found the number of dots to be particularly important (Benassi et al. 2010). While a wide range of parameters was coded in the current study, not all parameters were included in the analysis. For some parameters not enough information proved available (information not reported in papers) in order to be included in the analysis. Examples of these include dot size, dot density and central versus peripheral stimulus presentation. Stimulus duration and motion speed (for both biological motion and coherent motion) and number of dots (for coherent motion) were considered valuable and retained for further analysis.

With regard to participant characteristics, mean chronological age, mean intellectual abilities and type of group-wise matching were included for further analysis. Previous studies have suggested that participant characteristics such as age, verbal and non-verbal ability, and language delay may contribute to differences in performance on motion processing tasks (Jones et al. 2011; Koldewyn et al. 2010; McKay et al. 2012; Rutherford and Troje 2012; Takarae et al. 2008). However, the question remains whether a difference in performance is (more) influenced by maturation (i.e., slower development with age in ASD) and/or cognitive ability (i.e., discrepant IQ profiles in ASD). In addition, participant characteristics may prove particularly relevant given motion thresholds are known to change throughout development (Hadad et al. 2011; Hadad et al. 2015).

### Data Analysis

For each observation, using the descriptive statistics or test statistics (e.g., *t*- or *r*-values) present in the included papers, we calculated Hedges’ *g* as the estimate of the difference in population means divided by the common standard deviation. A standard correction to Hedges’ *g* was applied to account for a bias for small sample size (Hedges 1981). In addition, we estimated the standard error $\sigma_{g}^{ }$ of each observation, to determine the weight of each effect size and to estimate the precision of the estimates of the parameters of our meta-analytic model. According to our model, Hedges’ *g* is negative when the ASD group is outperformed by the typically developing group and positive when the ASD group outperforms the typically developing group. According to the guidelines of Cohen (1988), an absolute effect size of 0.2 to 0.3 is regarded as a small effect, around 0.5 as a medium effect and from 0.8 on as a large effect.

All analyses were conducted with Hedges’ *g* as the dependent variable. Each individual effect size was weighted by the estimated precision, the inverse of the (estimated) variance of the effect size estimate. Because the data of most studies resulted in more than one effect size, a traditional (two-level) random effects model was extended to a three-level random effects model (Van den Noortgate et al. 2013). Henceforth, random sampling variation of observed effect sizes, variance between outcomes studied within the same study, and between-study variance are taken into account. This three-level model is a linear model that entails a residual term for each kind of variance. The simplest model, a model without moderator variables, is given in the Eq. 1:

1 $${\text{g}}_{\text{jk}} = \beta_{0} + {\text{v}}_{{ . {\text{k}}}} + {\text{u}}_{\text{jk}} + {\text{e}}_{\text{jk}}$$

where *g*_*jk*_ is the observed effect size for outcome *j* within study *k*; *β*_0_ is the overall mean effect size, across all outcomes and studies. Element *v*_.*k*_ refers to the random deviation of the (mean) effect in study *k* from the overall effect over studies, *u*_*jk*_ to the deviation of the effect for outcome *j* in study *k* from the mean effect in study *k*, and e_*jk*_ is the residual due to sampling fluctuation, indicating the deviation of the observed effect size from the population effect size for outcome *j* in study *k*. All three residuals, *v*_*.k*_*, u*_*jk*_*, e*_*jk*_ are assumed to be independently normally distributed with zero mean. Because the sampling variance (i.e., the squared standard error) for each *g*_*jk*_ has been estimated using reported data before conducting the meta-analyses, only the mean effect size *β*_0_, the between-study variance $\sigma_{v}^{2}$ and the within-study variance $\sigma_{u}^{2}$ are estimated in the meta-analysis. This model was extended by including each of the coded study characteristics as predictors in separate models, as in ordinary regression models, to investigate their impact.

Parameters of the three-level meta-analytic models were estimated using the restricted maximum likelihood estimation, implemented in the mixed procedure of the general statistical software package SAS, Version 9.4 of the SAS System for Windows (SAS University Edition 2013). All significance tests were conducted with a significance level of 5%.

## Results

This meta-analysis examined 48 articles: 20 articles (127 effect sizes) which related to biological motion and 28 articles (100 effect sizes) which related to coherent motion. Mean age of participants ranged from 6 to 37 years (*M* = 21, *SD* = 7), gender ratio from 0% to 57% females (*M* = 11%, *SD* = 13%), and mean full scale IQ from 42 to 125 (*M* = 111, *SD* = 9). The total sample size (across participant groups) ranged from 18 to 141 per study (*M* = 34, *SD* = 13).

A random effect analysis of the overall effect size revealed a mean estimate of − 0.30, with 95% confidence limits from − 0.17 to − 0.44, *t*(36.8) = − 4.47, *p *< .0001. In line with Cohen’s (1988) guidelines, this effect is regarded as a small mean effect. Overall, participants with ASD were less accurate, slower or needed more information when detecting or discriminating global motion patterns, relative to participants without ASD. Both the between-study variance $\sigma_{v}^{2}$ (estimate = 0.12, *z* = 2.72, *p* = .003) and the within-study variance $\sigma_{u}^{2}$ (estimate = 0.07, *z* = 3.04, *p* = .001) were significant, suggesting that effect sizes varied significantly both across and within studies.

### Publication Bias

**Fig. 2:**
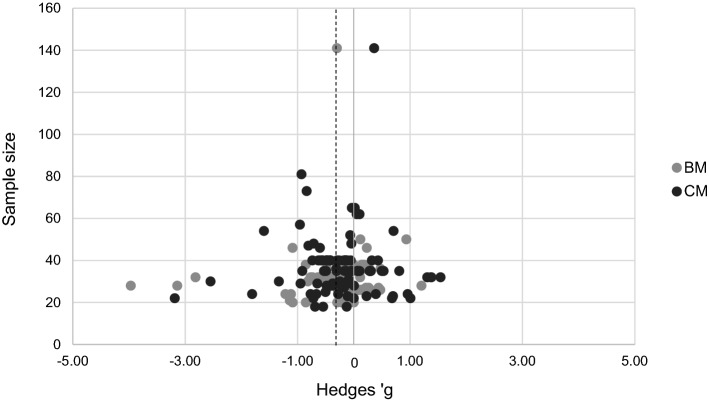
Funnel plot of the 227 effect sizes (Hedges’ g) as a function of the sample size. Negative effect sizes indicate worse performance for individuals with ASD compared with the TD group. The dotted line represents the mean estimated effect size, − .30, while the full line represents a mean estimated effect size of zero. *BM* biological motion, *CM* coherent motion

As a first evaluation of publication bias, each observed effect size was plotted against its corresponding sample size in a classic funnel plot (Egger et al. 1997). In the absence of publication bias, one can expect studies to be distributed symmetrically around the mean effect size. By contrast, in the presence of publication bias, one can expect the bottom of the plot to show a higher concentration of studies on one side of the mean than on the other, indicating that smaller studies are more likely to be published if they have larger than average effects (Cooper 2009). For the current data, the funnel plot shows a slight asymmetry to the left, which could be an indication of publication bias (Fig. 2) in favor of a global motion deficit for individuals with ASD. However, as a funnel plot merely offers a visual sense of the relation between the observed effect size and the corresponding sample size and its interpretation is largely subjective, a Begg and Mazumdar (1994) rank correlation test was performed in an attempt to quantify the amount of bias captured by the funnel plot. If asymmetry is caused by publication bias one would expect high standard errors (small studies) to be associated with larger effect sizes. For the current data, however, Kendall’s tau correlation coefficient, used to assess Begg and Mazumdar’s rank correlation test, revealed no correlation between the sample size and the observed effect size, т = .006, *p* = .89, indicating there is no true publication bias present in the data. This is not surprising, given the fact that global motion research in ASD is known for its mixed results, making all data, both significant and non-significant results in either directions, likely to be considered valuable and accepted for publication to equal extents.

### Moderator Analysis

#### Task Characteristics

With regard to task characteristics, the type of paradigm (biological motion vs. coherent motion), the type of task (motion discrimination vs. motion detection) and type of dependent variable (accuracy, RT data, threshold or d-primes) were evaluated. To examine whether the type of global motion paradigm influenced the size of the group difference between the ASD and TD group, we compared the mean effect size for biological motion and coherent motion. Yet, this analysis revealed no significant moderator effect, *F*(1, 56) = .29, *p* = .59. Mean effect size estimates for biological motion (*M* = − .26; CI − 0.06 to − 0.46) and coherent motion (*M* = − .33; CI − 0.16 to − 0.51) did not significantly differ, indicating that global motion differences between participants with and without ASD are of comparable size for both paradigms. To examine whether the type of global motion task influenced the size of the group difference between the ASD and TD group, we compared the mean effect size for detection vs. discrimination tasks. This analysis, however, revealed no significant moderator effect, *F*(1, 68) = .72, *p* = .40. Mean effect size estimates for global motion detection (*M* = − .26; CI − 0.07 to − 0.44) versus global motion discrimination (*M* = − .36; CI − 0.17 to − 0.55) did not significantly differ, indicating that global motion differences between participants with and without ASD are of comparable size for both task types. To examine whether the type of dependent variable influenced the size of the group difference between the ASD and TD group, we compared the mean effect size for accuracy, RT, threshold and d-prime data. However, comparing these mean effect size estimates, the analysis revealed no significant moderator effect, *F*(3, 158) = .73, *p* = .53. A negative mean effect size was revealed for each of the four dependent variables (*ps *≤ .005) but all four were comparable in size, or at least, not significantly different. Note, that while these last two analyses were conducted for biological and coherent motion data combined, neither of these analyses produced formally different results when ran for each paradigm separately.

#### Stimulus Characteristics

As for stimulus characteristics, stimulus duration and motion speed (for both biological motion and coherent motion) and number of dots (for coherent motion) were evaluated. To examine whether the stimulus durations used in global motion paradigms influenced the size of the group difference between the ASD and TD group, we included stimulus duration as a continuous variable, ranging from 160 ms to 6500 ms. Yet, this analysis revealed no significant moderator effect, *F*(1, 35.2) = 0.06, *p* = .80. Again, while this analysis was conducted for biological (range 540 to 6500 ms) and coherent motion (160 to 6000 ms) data combined, this analysis did not produce formally different results when ran for each paradigm separately.

To examine whether the speed of motion used in global motion paradigms influenced the size of the group difference between the ASD and TD group, we included motion speed as a continuous variable, ranging from 2.0 deg/s to 12.0 deg/s. However, this analysis produced no significant moderator effect, *F*(1, 37.9) = 0.95, *p* = .34. While motion speeds were available for most of the coherent motion data, they were less often available for biological motion data. We therefore confirmed that running the analysis on motion speed for coherent motion data separately did not produce formally different results. To examine whether the number of dots used in the coherent motion influenced the (size of the) group difference between the ASD and TD group, ranging from 100 to 2000. Again, this analysis revealed no significant moderator effect, *F*(1, 42.4) = 4.78, *p* = .63. Note that the number of dots used in biological motion tasks was not included in the analysis because it does not vary as much as in coherent motion paradigms.

#### Participant Characteristics

With regard to participant characteristics, mean chronological age, intellectual ability and group matching were evaluated. To examine whether the participants’ chronological age influenced the (size of the) group difference, we included mean age as a continuous variable. Comparing global motion studies across development, no significant moderator effect was revealed, *F*(1, 37.7) = 1.17, *p* = .29. To examine whether participants’ intellectual abilities influenced the size of the group difference, we included mean VIQ, mean NVIQ and mean FSIQ as continuous variables. However, none of these analyses revealed a significant moderator effect, (VIQ: *F*(1, 15.9) = .04, *p* = .85; NVIQ: *F*(1, 16.9) = 2.14, *p* = .16; FSIQ: *F*(1, 23.4) = 1.36, *p* = .26). Finally, we examined whether matching both groups on IQ influenced the size of the group difference between the ASD and TD group, comparing effect sizes for groups matched on IQ (i.e. VIQ, NVIQ and/or FSIQ) with groups not matched on IQ. Yet, this analysis revealed no difference in the size of the group differences, *F*(1, 58.8) = .93, *p* = .34.

## Discussion

Potential deficits in global motion perception in individuals with ASD have long been a topic of intense investigation. The conclusions to be drawn from this literature, however, have remained unclear, with a wide variety of differences in task, stimulus and participant characteristics as well as divergence in study findings. In this paper, we conducted a meta-analysis to examine whether individuals with ASD differ in global motion processing, investigating both biological motion and coherent motion, compared to TD individuals. Our results reveal a small mean effect of − 0.30, with 95% confidence limits from − 0.17 to − 0.44, indicative of global motion processing difficulties in ASD. This mean negative effect was apparent for both biological motion as well as coherent motion paradigms. None of the potential moderators that were evaluated proved essential in understanding performance differences between both groups.

### Moderators of the Effects

First of all, our results revealed a mean effect of − 0.30, indicating that overall participants with ASD were less accurate, slower or needed more information when detecting or discriminating global motion patterns, relative to TD participants. In line with Cohen’s (1988) guidelines, a mean effect of − .30 is regarded as a *small* effect. Similar size mean effects have been found for other aspects of visual functioning in ASD, for instance, local–global visual processing of static stimuli (*ES* = − 0.23; Van der Hallen et al. 2015) and visual orienting in Posner-like tasks (*ES* = − 0.44; Landry and Parker 2013). In order to detect small effects, large sample sizes (*N* > 200) are required, if not, the effect will not be revealed in a consistent manner and the literature as a whole will become subject to inconsistencies (G*Power; Faul et al. 2007). As all researchers are limited in the amount of time and participants they can secure for individual studies and large samples sizes are required if one wants to detect similar small effects, collaborative efforts should be welcomed in future to increase the number of participants and to avoid new underpowered studies on visual processing in ASD.

To further examine the mean difference in global motion processing between individuals with and without ASD that was revealed, a number of potential moderators were investigated. Despite the wide range of potential moderators that were investigated, no significant main effects were found. This is somewhat surprising, given the substantial (and significant) between- and within study variability.

With regard to task characteristics, the type of paradigm (biological motion vs. coherent motion), type of task (detection vs. discrimination) and the type of dependent variable were evaluated. The importance of the type of paradigm, i.e. biological motion versus coherent motion, has been argued for previously in an attempt to unravel the mixed evidence in the current literature. When reviewing the literature, Kaiser and Shiffrar (2009) noticed that, while initial findings on coherent motion seemed promising (in finding pronounced group differences), later findings seemed to indicate that deficits in global motion processing in ASD were limited to biological motion, and not seen so much for coherent motion. In addition, it has been suggested that while both paradigms rely on the integration of local motion information into a global motion pattern, the social nature of biological motion might make it more difficult for individuals with ASD, and for that reason, may elicit greater group differences (e.g., Koldewyn et al. 2010). Yet, when we compared the size of the group difference between the ASD and TD group for biological motion vs. coherent motion paradigms, no significant difference was revealed. Note, however, that data which focused on the ability to extract social or emotional content from motion displays (e.g., Hubert et al. 2006; Moore et al. 1997; Nackaerts et al. 2012; Parron et al. 2008) were not included in the analysis. In line with the fact that the type of paradigm did not have pronounced moderating effects, group differences were also not impacted differently depending on the type of task (i.e. motion detection vs. motion discrimination) or the type of dependent variable (i.e. accuracy, RT, d-prime or thresholds). While the potential importance of the type of task, i.e. motion detection versus motion discrimination, has not received particular attention, one could easily argue that detecting some global structure in a range of motion patterns is a more low-level task than discriminating or identifying a range of specific global motion patterns or more high-level attributes specified by them. As discussed, previous research has already suggested that different mechanisms and different neuronal populations are involved in motion detection compared to motion discrimination (Hol and Treue 2001; Koyama et al. 2010). However, comparing performances for motion detection and motion discrimination tasks, no differences between both tasks were revealed. The type of dependent variable has proved important in a previous meta-analysis on visual processing in ASD using static, non-social stimuli, where RT was more important than accuracy (Van der Hallen et al. 2015). Yet, when evaluating the size of the group difference for the different dependent variables with regard to global motion processing, no difference was revealed. It is difficult to speculate as to why the dependent variable proved important in the meta-analysis on local versus global processing of static stimuli but not in the current meta-analysis on global motion perception tasks, as there are a number of similarities and differences, however, it is tempting to presume that the intrinsically dynamic nature of motion has something to do with it.

Mirroring the lack of moderating effects of more general task characteristics, no moderating effects for the stimulus characteristics were revealed. As mentioned, previous research in ASD has revealed interesting results with regard to stimulus duration (Robertson et al. 2012; but also see Davis et al. 2006), presentation location (Ronconi et al. 2012) and motion speed (Manning et al. 2013). In addition to that, research with regard to typical development has suggested that dot lifetime, speed and density are important parameters (Hadad et al. 2015, although see 2011). Nevertheless, in our analysis no moderating effect of stimulus duration, motion speed or number of dots was revealed. One possibility is that these factors are in fact not important when it comes to group differences in motion processing in individuals with ASD relative to TD individuals. Another possibility, however, is that their potential effect was clouded by pooling the data across studies which differed on a number of other characteristics (i.e., overshadowing the effect), while complex unknown interactions could be at play. For instance, while no effects of motion speed where revealed, future investigations focusing on spatial displacement may still reveal important effects. Unfortunately, only a few studies exist which have actively investigated a particular task or stimulus characteristics within one and the same group, making it difficult to otherwise assess the potential impact of the parameters that have been investigated. In light of that, we would argue that to further evaluate the effect of these stimulus parameters, more within-participants designs are needed which actively investigate a particular parameter while controlling for all other variables (same participants, same age categories, same IQ abilities, same task, same setting, etc.).

Finally, participant characteristics such as the mean chronological age, mean intellectual ability and type of group matching were evaluated as potential moderators. To start, it is important to note that nearly all previous global motion studies in ASD have taken chronological age and/or the mental age of both participant groups into account. This is imperative, as it has been found that, depending on the speed, motion thresholds can change during development (Hadad et al. 2015, however, Hadad et al. 2011). Whereas early studies on global motion processing in ASD focused largely on children and/or adolescents, recently, more studies have started to include adult populations. Some of the studies have found a link between intelligence and global motion and/or have argued that individuals with higher levels of intellectual functioning may be better equipped to develop compensatory strategies to reach equal performance levels via routes that are bypassing or modulating the default perceptual modes of processing (Atkinson 2009; Koldewyn et al. 2010; Rutherford and Troje 2012). Nevertheless, a large number of studies have not found a relation between either verbal or nonverbal IQ and global motion performance (e.g., Parron et al. 2008). In line with that, the results of our meta-analysis suggest that differences in global motion perception between individuals with and without ASD are not strongly linked to chronological age or mental age, as neither age, nor (matching on) intellectual abilities proved to be an important moderator in ruling the variability.

As this meta-analysis did reveal significant within- and between-study variability, but failed to identify significant moderating variables, one has to wonder whether a large amount of individual variability, not accounted for by chronological age or IQ, might be at play and might cloud these results. In fact, one of the most pertinent features of motion data, for both participants with and without ASD, is the large amount of individual variability in motion thresholds (Hadad et al. 2015; Manning et al. 2014). With such inter-individual variability, it is likely that some degree of overlap between both participant groups exist, hindering the more-detailed moderator analyses and our understanding of differences. Future research will be needed to understand what factors contribute to this large inter-individual variability in motion processing.

### Understanding Task Impairment in ASD

With results indicating a small, though statistically significant global motion processing impairment in ASD, one is left to wonder what is actually driving this difference. One possibility could be that these differences between individuals with and without ASD in global motion processing are unrelated to motion processing, but domain-general differences in task motivation, attentional abilities or visual integration skills are at the core of this. If participants with ASD are less motivated to participate or struggle more to attend to the motion stimuli, this might explain these group differences. However, researchers have gone to considerable lengths to ensure that their participants engage in the task and remain motivated by using game-like scenarios (e.g., Manning et al. 2013), capturing sounds (e.g., Del Viva et al. 2006), catch trials (e.g., Manning et al. 2013; Milne et al. 2006), or control attention using eye-tracking measures (e.g., Manning et al. 2015). Moreover, some researchers have revealed group differences to be specific to motion coherence tasks as opposed to other visual (integration) tasks, such as form-processing tasks (e.g., Spencer et al. 2000; but see Milne et al. 2006). While we cannot rule out this hypothesis based on the results of the meta-analysis, the existing evidence does suggest other, motion-related causes to be at play.

Currently, the most popular interpretation of global motion processing difficulties in these tasks, is that of atypical integration of motion signals in individuals with ASD (Annaz et al. 2009; Frith and Happé 1994; Gepner and Mestre 2002; Manning et al. 2013; Pellicano et al. 2005; Robertson et al. 2012). That way, motion perception in individuals with ASD is not inherently atypical, but their *integration* of local motion signals into a global motion percept operates differently. What speaks in favor of this idea of atypical integration, is the fact that individuals with ASD have shown typical or enhanced performance on motion tasks that did not require integration of local motion cues (e.g., Bertone et al. 2003, 2005; Foss-Feig et al. 2013; Pellicano et al. 2005) and have shown elevated motion coherence thresholds for stimuli presented at short but not long durations, suggesting individuals with ASD need more time to properly integrate the information (Robertson et al. 2012; but also see Davis et al. 2006). However, the standard global motion paradigms do not provide a pure measure of motion integration abilities, as the ability to integrate information is confounded by the ability to perceive local motion and the ability to segregate signal from noise.

Individuals with ASD may show difficulties in global motion processing due to a reduced ability to estimate the local motion of each dot, which could be a consequence of increased internal noise (neural variability) in individuals with ASD (Simmons et al. 2009). Evidence for increased internal noise in ASD has been found in a number of neuroimaging studies, revealing increased trial-by-trial variability or spontaneous fluctuations in neural activity (e.g., Milne 2011; Pérez Velázquez and Galán 2013). However, more recently Davis and Plaisted-Grant (2015) argued these results to be inconsistent with *increased* internal noise, and argued *reduced* internal noise in ASD instead. Alternatively, it has been suggested that difficulty with motion perception is due to a poor ability to segregate signal dots from masking noise dots (Manning et al. 2015; Van de Cruys et al. 2016; Zaidel et al. 2015). The idea here is that individuals with ASD are less able, or less inclined, to segregate the signal from the noise, pooling all available “information” regardless of its nature. In most global motion tasks, segregating signal from noise is an important (implicit) part of the task at hand. Interestingly enough, some studies with noiseless motion paradigms (e.g., Chen et al. 2012; Foss-Feig et al. 2013) have shown enhanced motion perception in ASD.

Unfortunately, the standard coherent or biological motion paradigms cannot distinguish between local and global hindrance to global motion perception, and are uninformative with regard to the source of global motion difficulties in ASD (Dakin and Frith 2005). One way to investigate the source of global motion processing difficulties in ASD is to use an equivalent-noise paradigm (Barlow and Tripathy 1997; Dakin et al. 2005). This paradigm allows the relative contributions of local internal noise and global averaging ability on performance to be quantified, while removing the demand for segregating signal-from-noise. Whereas typical global motion paradigms contain both signal and noise dots, equivalent noise stimuli only contain dots with directions sampled from a single Gaussian distribution on a given trial (Dakin et al. 2005; Tibber et al. 2014). Rather than manipulating trial difficulty by adding incoherently moving noise dots, stimulus variability (i.e., external noise) is controlled by varying the standard deviation of the Gaussian of dot directions across trials. When an equivalent noise function is fitted to thresholds collected at varying levels of external noise, estimates of the individual’s internal noise and global averaging ability can be derived. Manning and colleagues have used such an equivalent noise paradigm to evaluate to what extent poor motion processing is due to local noise and/or poor averaging in both typically developing children (Manning et al. 2014) and children with ASD (Manning et al. 2015). Compared to typically developing children, the children with ASD showed typical levels of internal noise, but, surprisingly, *enhanced* integration of motion information. Yet, the children with ASD performed similarly to typically developing children in a standard motion coherence task. Taken together, these results argue against an explanation of global motion differences in terms of poor integration or an explanation in terms of atypical levels of internal noise, but rather suggest motion perception in ASD may be characterized by increased integration of motion signals in combination with reduced segregation of signal from noise.

Interestingly enough, difficulties in segregating signal-from-noise align nicely with more recent, domain-general accounts of ASD which have taken inspiration from information processing models of typical cognition (Lawson et al. 2014; Pellicano and Burr 2012; Van de Cruys et al. 2014). Particularly influential in these new proposals is the predictive coding framework (Clark 2013; Friston 2012), which assumes that our brains build models about the perceptual inputs it receives. While each of the new accounts suggests a slightly different model, the common ground is that predictions in individuals with ASD end up clouded by irrelevant information, biasing the generated predictions in faulty directions (see Van de Cruys et al. 2016 for further discussion).

Although a number of possible factors have been put forward which could constitute the underlying cause of these differences in global motion processing, it is clear that further research is necessary in order to move forward with this. As the standard motion coherence or biological motion paradigm do not allow to actively study the underlying factors, as has become evident from this discussion, others paradigms such as the equivalent noise framework should receive priority in going forward.

### Implications for Other Disorders

Atypical global motion thresholds have been reported in a range of developmental conditions, such as Williams syndrome (e.g., Atkinson et al. 2006; Reiss et al. 2005), Fragile X (e.g., Gallego et al. 2014; Kogan et al. 2004) schizophrenia (e.g., Chen et al. 2003; Kandil et al. 2013) and dyslexia (see Benassi et al. 2010 for a review). Consequently, it makes for an interesting question whether atypical global motion processing (and the underlying driving mechanism) is different for each of these developmental conditions or merely a general consequence of atypical development.

Pellicano and Gibson (2008) assessed the integrity of the dorsal visual pathway at lower subcortical (measuring sensitivity to flicker contrast) and higher cortical (measuring sensitivity to global motion) levels in TD children, children with ASD and children with dyslexia. While children with ASD demonstrated intact lower-level but impaired higher-level dorsal-stream functioning, children with dyslexia displayed abnormalities at both lower and higher levels of the dorsal visual stream, suggesting that these disorders can be dissociated according to the origin of the impairment along the dorsal-stream pathway (Pellicano and Gibson 2008). More recently, similar efforts were made by Tibber and colleagues using the equivalent noise paradigm. In a series of studies Tibber and colleagues investigated the factors underlying motion processing in schizophrenia (Tibber et al. 2015), individuals with migraine (Tibber et al. 2014), and ASD (Manning et al. 2015), revealing interesting differences. These results highlight the value of systematic cross-syndrome investigations of motion processing. To move forward in determining whether atypical motion relates to domain-specific versus domain-general development, cross-syndrome and cross-domain comparisons of full developmental trajectories are needed using appropriate motion paradigms (Annaz and Karmiloff-Smith 2005).

## Conclusion

This meta-analysis constitutes a first important step in understanding global motion processing in ASD, leading to three main conclusions. First, a small, though, significant overall group difference was revealed, indicative of global motion difficulties in individuals with ASD. Secondly, as none of the potential moderators proved informative with regard to group differences, global motion research should be contrasted more systematically with studies on basic motion processing, as well as focus more on within-study task manipulations, and avoid underpowered studies or between-study comparisons that are easily clouded by large inter-individual variability. Tasks such as the equivalent-noise paradigm, which allow for an active investigation of the factors potentially driving global motion impairments, should receive high priority. Last but not least, more efforts should be spent toward systematic cross-syndrome investigations of motion processing, given atypical global motion processing has been reported for a range of developmental conditions.
